# Perspectives of female sex workers on HIV pre-exposure prophylaxis delivery in Uganda: A qualitative study

**DOI:** 10.21203/rs.3.rs-4115528/v1

**Published:** 2024-03-21

**Authors:** Ruth Mpirirwe, Andrew Mujugira, Happy Walusaga, Florence Ayebare, Khamisi Musanje, Patricia Ndugga, Christine Muhumuza, Joan Nangendo, Fred C. Semitala, Peter Kyambadde, Joan Kalyango, Agnes Kiragga, Charles Karamagi, Moses R. Kamya, Mari Armstrong-Hough, Anne R. Katahoire

**Affiliations:** Makerere University; Makerere University; Makerere University; Infectious Diseases Research Collaboration; Makerere University; Makerere University; Makerere University; Makerere University; Makerere University; Ministry of Health; Makerere University; Infectious Diseases Research Collaboration; Makerere University; Makerere University; New York University; Makerere University

**Keywords:** FSW, HIV Prevention, Pre-Exposure Prophylaxis, Africa

## Abstract

**Background:**

HIV pre-exposure prophylaxis (PrEP) is underutilized by cisgender female sex workers (FSW) despite its proven effectiveness. This study aimed to understand the experiences of FSW with PrEP services in Uganda to inform HIV programming for this key population.

**Methods:**

We conducted qualitative interviews with 19 FSW between June and July 2022 at the Most at Risk Populations Initiative clinic, Mulago Hospital, Kampala, to explore experiences with accessing PrEP Indepth interviews explored: (1) descriptions of where and how PrEP was obtained; (2) perspectives on current approaches for accessing PrEP; and (3) individual encounters with PrEP services. Data were analyzed through inductive thematic analysis.

**Results:**

Three key themes emerged for FSW perspectives on PrEP service delivery. FSW highlighted the positive impact of a welcoming clinic environment, which motivated FSW to initiate PrEP and fostered a sense of connectedness within their community. They also reported feeling accepted, secure, and free from prejudice when accessing PrEP through facility-based services. The second explores the obstacles faced by FSW, such as lengthy wait times at clinics, inadequate provider support, and lack of sensitivity training which hindered their access to PrEP The third sheds light on how HIV-related stigma negatively impacted the delivery of community-based PrEP for FSW. While community-based distribution offered convenience and helped mitigate stigma, clinic-based care provided greater anonymity and was perceived as offering higher-quality care. Overall, FSWs emphasized the critical role of friendly healthcare providers, social support, and non-stigmatizing environments in promoting successful utilization of PrEP.

**Conclusion:**

The study findings offer insights that can support HIV programs in optimizing PrEP delivery for FSW. Establishing easily accessible drug pick-up locations, prioritizing privacy, addressing and improving health workers’ attitudes, and providing regular reminders could enhance PrEP access for FSW and decrease HIV acquisition.

## INTRODUCTION

Globally, there is a significant disparity in HIV prevalence between cisgender female sex workers (FSW) and women (aged 15–49) in the general population; FSW are 30 times more likely to be living with HIV^1^. This trend is also reflected in Uganda, where FSW comprise 18% of newly reported HIV cases and have high seroprevalence (31.3%)^2^. The Joint United Nations Program on HIV/AIDS (UNAIDS) global target is for 95% of people at risk of HIV to use person-centered and effective combination prevention methods like PrEP by 2025^3^. However, FSW encounter barriers at the individual, social and health system levels that hinder their ability to obtain appropriate HIV care^4^. At the individual level, factors such as frequent relocation, and alcohol or substance abuse contribute to marginalization and greatly diminish their agency and ability to engage and remain in care^5^. At the social level poverty, violence, healthcare system levels, disparities based on culture and economy, social stigma, governmental policies that criminalize sex work, and inadequate access to healthcare all contribute to higher HIV vulnerability^6,7^.

Daily oral pre-exposure prophylaxis (PrEP) is a highly effective strategy for preventing HIV acquisition^8,9^. While successful demonstration projects have shown the potential of PrEP for preventing HIV acquisition, effective PrEP use remains challenging in Uganda and other resource-limited settings, hindering prevention programs^10,11^. Better approaches to optimize PrEP delivery and scale-up are needed^12–14^ to ensure that PrEP is effectively utilized to prevent HIV.

Whereas differentiated PrEP service delivery is effective for adolescent girls and young women^15,16^, FSW experience unique challenges, including multiple sexual partners, inconsistent condom use, intersecting stigmas, discrimination by healthcare providers, limited access to education, inconvenient clinic operating hours, high costs of travelling to healthcare facilities, criminalization of sex work and high levels of alcohol and drug misuse^17^. This qualitative study aimed to explore FSWs’ experiences in accessing PrEP through existing community and facility PrEP delivery models in Uganda to better understand how well these models met their prevention needs.

## METHODS

### Study setting and design

The study was conducted at the Most At-Risk Populations Initiative (MARPI) clinic from 15th June to 17th July 2022. MARPI is located within the Mulago National Referral Hospital complex in Kampala, Uganda. The MARPI program was established in 2008, provides HIV care for over 20,000 FSW and other key populations (KP), and is funded by the U.S. Centers for Disease Control and Prevention (CDC). In August 2017, MARPI became Uganda’s first public health facility to offer KP free PrEP services. Care is delivered through a facility model that comprises of FSW receiving PrEP from MARPI clinic, and a community model where the PrEP health care providers who include PrEP counsellors, nurses, doctors, and laboratory technicians take the PrEP in central places in the communities where FSW can easily access the PrEP This study employed an exploratory qualitative design to explore and describe the experiences of FSW who utilized community and facility-based PrEP delivery models.

### Population and procedures

We purposively selected FSW for in-depth interviews by consulting PrEP counsellors at MARPI to identify FSW who had initiated PrEP and attended refill visits (the phenomenon of interest) and met the following criteria: 1) willing to provide informed consent to participate in the study, 2) at least 14 years of age (national guidelines permit informed consent by emancipated or mature minors)^18^, and 3) had been on PrEP for at least one year or started but discontinued within one year. Participants who were involved in another PrEP or HIV prevention study, those with severe illness, allergic to tenofovir disoproxil fumarate (TDF), lamivudine (3TC), emtricitabine (FTC), or other PrEP drug, individuals with hepatitis B (confirmed through self-report or medical records), and those with chronic kidney disease (ascertained through self-report or medical records) were excluded from the study.

FSW peers invited participants to a study information session at the MARPI clinic. The study staff introduced themselves to the participants and explained the purpose of their visit. To capture a diverse range of FSW experiences, we utilised the maximum variation approach, which included factors such as age, marital status, and educational background. This method allowed a sample with varied characteristics, translating into variations in PrEP delivery experiences.

### Data collection

Two experienced female interviewers with a Bachelor’s degree and fluent in Luganda and English conducted the interviews based on the language preferences of the FSW. The interviewers were trained on the interview guide before collecting the data. The interviewers used a semi-structured interview guide with questions guided by the Social Ecological Model^19^ to gather FSW opinions, suggestions, and perspectives regarding their experiences with PrEP access. The topics discussed included: (a) sources and frequency of obtaining PrEP (b) views on the current delivery models for accessing PrEP and (c) personal accounts of using the existing PrEP delivery model. Interviews were conducted in a designated room at the MARPI Clinic and lasted about 60 minutes. All interviews were audio-recorded with permission. We stopped data collection when we reached data saturation at the 19^th^ interview^20^, indicating that no further insights were being obtained. A linguistics expert from Makerere University Institute of Languages transcribed all the Luganda audio recordings verbatim, cross-referencing the transcripts with the original recordings and field notes. A bilingual research assistant translated transcripts into English by reading and understanding the source text, keeping the meaning in mind, and selecting the most appropriate vocabulary in English. To ensure the safety and confidentiality of our study documents, we securely stored audio files on an encrypted hard drive and uploaded them to the server weekly. Paper documents, including consent forms, were kept in a locked location in the Makerere Behavioral and Social Sciences Research project office.

### Trustworthiness

This study acknowledged the pivotal role of translation in shaping knowledge and emphasized the active involvement of translators as agents in the research process. As such, translators were well-versed in the theoretical framework and objectives of the research. The translator used a meaning-based approach to translate from Luganda into English. The primary goal was to accurately convey the intended meaning of the source language while adhering to the target natural grammar of English.

### Quality control

We clarified text in square brackets to capture and interpret meaningful elements of the source material for the reader and how the elements combined to form the meaning of the text. The translation quality was evaluated based on comprehensibility (especially relating to culture-specific concepts), appropriateness in content and approach, and accuracy in remaining faithful to the source text and key facts. Another field team double-checked text paragraphs to ensure fidelity and appropriate communication of meaning. Recordings were stored on a secure password-protected computer, accessible only to research staff.

### Data analysis

We used an inductive analytic approach to data analysis^21^. This flexibility allowed the data to guide the team’s analysis and identify emerging concepts. Authors RM, FA, HAGW, and KM daily reviewed interview transcripts through an iterative process for identifying content on PrEP access experiences. They also performed open coding on nine transcripts to identify specific text sections by outlining and provisionally labelling relevant content. The codes were defined, discussed, and arranged under emerging concepts, and after that, a codebook was developed, which was applied to the remaining transcripts. We coded the data using Atlas.ti (version 22), extracted quotations and synthesized them. On completion of the coding process, we used queries to sort the data and identify themes corresponding to PrEP access experiences. Each theme is presented in Results through a descriptive label, elaborative text, and interview quotes illustrating the theme.

### Ethics approval

The School of Medicine Research Ethics Committee of Makerere University College of Health Sciences (Mak-SOMREC-2022–299) and the Uganda National Council for Science and Technology (SS1223ES) approved the study. We obtained administrative clearance from Makerere University’s Clinical Epidemiology Unit and Mulago National Referral Hospital Ethics Committee. We obtained written informed consent from the study participants by the principles of Good Clinical Practice. We thoroughly explained the study’s objectives, benefits, and possible risks to all participants^22^. Additionally, we emphasized that participation was entirely voluntary, interviews would be audio recorded for accuracy, and they retained the right to withdraw from the study without justification. Anonymity and confidentiality were maintained by de-identifying the data. Each participant received an IRB-approved reimbursement of 20,000 Uganda Shillings ($5.30) for their time, effort, and transport costs.

## RESULTS

We screened 30 FSW for eligibility and interviewed 19. Eleven were ineligible; ten had discontinued PrEP > 1 year ago, and one declined to participate. The median age was 24 years (interquartile range [IQR] 21 – 32). Eighteen FSW (95%) were not married, 11 (58%) achieved primary-level education, and 8 (42%) had taken PrEP for more than one year (Table 1).

### Qualitative results

Three themes emerged from FSWs’ descriptions of experiences with PrEP service delivery in facility and community settings. The first theme describes the positive impact of a welcoming clinic environment, which motivated FSW to take PrEP and fostered a sense of connectedness within their community. The second explains how barriers faced by FSW, including long wait times at clinics and a lack of support from providers, led to difficulties in accessing PrEP. The third theme highlights how HIV-related stigma negatively affected PrEP delivery for FSW. Overall, FSWs pointed to friendly providers, social support, and a non-stigmatizing environment as crucial in promoting their PrEP utilization.

### Theme 1: A friendly and welcoming service environment facilitated PrEP uptake

FSW reported that the MARPI clinic was a welcoming, inclusive and safe space, encouraging them to continue utilizing its services. Furthermore, the staff at the clinic were described as friendly and non-judgmental, creating a comfortable environment for the women. Privacy at the facility also played a crucial role in reducing the stigma of being HIV-positive and provided much-needed social support. Overall, most FSW had positive experiences with health workers at the clinic. FSW reported that health workers displayed good humor and provided effective counselling.

“It is convenient to come to the facility because no one knows me here, and thus, I feel safe picking my drugs from here. The people here are welcoming and not judgmental. They welcome you the way you are. They do not segregate between the rich and the poor. I observed my fellow FSW and the doctors’ cooperation. If you ask for a doctor and that doctor is not there, they give you a seat as you wait for him. So, if I was mistreated, I couldn’t have returned”

(FSW, age 25)

“I won’t lie to you; nurses here are the most disciplined and with a sense of humility. I even know them all by name. They have become my friends. So far no one has made things complicated for me. You would come, hand in your card, and get your medicine”.

(FSW, age 41)

FSW reported that they had formed friendships with other sex workers at the clinic, which allowed for information sharing and learning from their peers. They developed strong bonds through phone conversations, including sharing thoughts on education, religion, and healthcare. These interactions fostered a sense of community connectedness among FSW, who supported each other by attending PrEP appointments together despite having different scheduled dates. However, some FSW felt more supported by male health workers compared to their female counterparts, whom they perceived as gossiping about their personal health information and breaching confidentiality, which negatively impacted the clinic visit experience.

“I have made friends with other sex workers at this facility. We are always there for each other. When the nurse realises we are all around, they feel happy and empowered. We tell them we shall go back [for PrEP]. We call ourselves over the phone and discuss school, church, and medicine. We became like family. We don’t have similar appointment dates, but sometimes we accompany each other.”

(FSW, age 19)

“… The ladies [women providers]gossip a lot. When you come to pick up PrEP and tell them you have an STI, they will give you treatment, but once you leave, they start talking about you, saying do you see that one in a jacket? She is infected with STIs. We keep telling them to use condoms, but they don’t.”

(FSW, age 21)

### Theme 2: Inadequate provider support and long clinic waiting times impeded PrEP access

FSW expressed discontent with the limited counselling services offered during community outreach visits by young, inexperienced nurses, who were new to key population programming. These nurses seemed more focused on identifying new cases of HIV than providing adequate PrEP support for those without the virus. FSW felt the clinical officers and nurses needed sensitivity training to reduce stigma and encourage the provision of non-judgmental health care. They perceived such health workers as insensitive [uncaring], pointing fingers and saying that they [FSW] were prostitutes. The FSW community attributed their forgetfulness in taking pills to the poor quality of counselling and, therefore, required consistent reminders from healthcare professionals to adhere to PrEP.

“Hmmmm… the young nurses they send into the community don’t know how to counsel people. They test and when they find you are not infected, they give you PrEP. They move with PrEP when they come to test. Some just come to find a positive client. When they don’t get any they leave annoyed. And you know for us sex workers, we like being pampered.”

(FSW, age 38)

“I realised they [doctors] don’t care. Those doctors need training on Nneeko [FSW] activities. They would point at you, saying there is a prostitute; she has come for drugs. When I heard that, I felt bad. I would leave the place [community]. You see, when you’re taking that drug, it needs someone to keep reminding you and being tolerant because you are tired, and by the time you remember, they have neglected you.”

(FSW, age 32)

FSW reported that the facility’s clinic flow caused dissatisfaction due to delays, such as waiting in line for blood draws, receiving test results, and then having to queue again to see health workers for their prescriptions and to receive PrEP at the pharmacy. These delays had a negative impact on the overall experience of receiving PrEP care.

“Only the line [queue] issue. Go here and there delays us. I don’t deny blood testing because you may take the medicine when the nurse isn’t sure about [your] status, and how does the nurse know? It’s out of the blood test. You sit there, join the line, and then undergo blood testing. That’s why they [FSW] complain about the time. You may return from the blood test, and the nurse will send you to another line. You will leave this place at around 2 pm”.

(FSW, age 41)

### Theme 3: HIV-related stigma hampered PrEP delivery

FSW preferred that PrEP be delivered in the community because of conflicting work and clinic schedules and a desire to avoid stigma associated with receiving PrEP at the facility. Sex workers reported that alcohol consumption, night shifts, and daytime sleeping schedules made it difficult for them to obtain PrEP from healthcare facilities due to inflexible clinic operating hours. As a result, they expressed greater comfort with receiving PrEP in their community, including at the local bars where they worked. This approach helped alleviate the stigma they often experienced when obtaining the medication at a health facility. They believed that accessing PrEP within their community spared them from potential judgment or negative assumptions about their HIV status, considering that they were using antiretroviral drugs.

“Some sex workers get drunk, and by the end of the day we have not gone for the medicine from the clinic. But that’s okay when they bring the medicine to the bar at around 6 am. You hear someone say let me sleep for about thirty minutes, and I go to Mulago. By the time she wakes up, it’s 1 pm. But it would be better to bring PrEP nearer, like in the bar”

(FSW, age 41)

“I am comfortable picking my PrEP drugs from the community because no one will see the pills and start accusing me of [being] HIV-positive. There was one time I went to pick up the drugs from the facility with a friend of mine who is also my client, and when he saw the drugs, he started accusing me of infecting him with HIV. I told him I was not sick, but he refused [to believe me]”.

(FSW, age 20)

While PrEP delivery in the community addressed the issue of stigma in healthcare facilities, it did not eliminate HIV-related stigma. This was particularly evident when other individuals witnessed healthcare workers providing medication to sex workers. To maintain confidentiality, nurses dispensed medication in unmarked clinic vehicles away from public view. Despite these precautions, some FSW preferred receiving PrEP services at clinics that offered greater anonymity and perceived higher quality of care compared to community settings. One FSW recounted, “… *switch[ing]back to getting PrEP from the facility because the healthcare workers in the community didn’t care”*.

“They can’t give you the drugs when people are seeing you in the community. They can test you, then call you to their car and give you the medicine, and you put it in your bag. When people see you, they might think you are just having an HIV test ”

(FSW, age 18)

“I wish the medicine [PrEP] stayed at the facility. Because the villagers may start talking about us. There’s somewhere I was in Gayaza, and they brought for me medicine, and my landlord saw them, laughed at me, and started telling others that I am HIV-positive”.

(FSW, age 24)

## DISCUSSION

Our study found that a welcoming and supportive clinic environment played a crucial role in facilitating PrEP use in this sample of Ugandan FSW. However, they preferred male health workers over females due to concerns about the confidentiality of their personal health information. We identified lack of provider support, negative attitudes of healthcare providers and long waiting times at clinics as significant barriers to accessing PrEP Some FSW preferred community-based PrEP delivery over clinic-based care due to its ability to avoid the interpersonal stigma commonly experienced in healthcare facilities. Despite this benefit, community delivery did not eliminate HIV-related stigma from intimate partners and the public—and some FSW felt that clinic-based delivery sheltered them from stigma more effectively than community delivery.

FSW reported that a welcoming environment encouraged them to continue utilizing its services. This finding is similar to research from Senegal which found high interest and good PrEP retention among FSW who received PrEP from Ministry of Health clinics^23^. Studies have shown that FSW appreciate PrEP introduction within familiar and trusted “friendly” services tailored for sex workers and value positive encouragement from clinic staff and perceived good quality of health services with same-day results^24–26^ The community connectedness we observed is consistent with other research conducted in Uganda, which also found that FSW actively supported one another in seeking medical care^17^. This social support has been shown to significantly impact the uptake of prevention methods^27^.

Facility-based care was a source of dissatisfaction due to delays, such as waiting in line for phlebotomy, receiving test results, and queuing again to see health workers for counselling and prescriptions. Other research has shown that FSW face challenges in unsupportive community environments. Long wait times, particularly in static clinics where services are provided alongside the general population, hinder their enrollment and retention in HIV care^28,29^. Our findings are consistent with a study of FSW perceptions and experiences accessing HIV services in 12 districts of Uganda, which revealed significant concerns about service quality, including discrimination and disrespectful comments from providers, refusal or delay of services, and potential breaches of confidentiality^30^. To enhance PrEP retention among this population group, decentralizing PrEP services and providing sensitivity training for providers could be beneficial.

FSW favored community PrEP delivery due to conflicting schedules and a desire to avoid stigma. The results of this study highlight the convenience of the community-based care in providing high-quality services that help circumvent HIV-related stigma and promote person-centered care, resulting in improved retention in PrEP care^31^. Utilizing peer sex workers as PrEP providers is essential, as previous research has shown their ability to effectively relate to and support fellow sex workers^25,30,32–34,35,36^. FSW may experience both internalized and anticipated stigma, leading to feelings of shame, fear, and low self-esteem. FSW also struggle with the social stigma associated with their occupation, which can lead to concerns about visiting clinics and potential consequences if their identity as a sex worker is revealed^37^. Implementing multi-level interventions targeting the intersecting stigmas faced by FSW can potentially enhance adherence to PrEP and retention in healthcare for this population^38^.

Our study’s strengths include qualitatively evaluating facility and community-based PrEP delivery within the national PrEP program. We purposively sampled FSW respondents and used maximum variation to avoid potential selection bias. The limitations of this work include social desirability and recall bias, which may have influenced the findings. Since FSW were purposefully sampled, this may have affected analytical results. Furthermore, the study conducted in one geographic setting (Kampala) may not accurately reflect the PrEP experiences of all FSW in Uganda. However, they may provide valuable insights for the implementation of PrEP as a biomedical HIV prevention intervention in similar settings.

## Conclusions

This study suggests that to ensure optimal utilization of PrEP among FSW, it is essential to tailor PrEP delivery to meet their specific preferences and needs. This can be achieved by offering multiple access points for PrEP and providing sensitivity training for healthcare personnel. Integrating community-based PrEP into existing national programs can serve as an incentive for successful implementation and contribute towards meeting both national and global targets for HIV prevention.

## Figures and Tables

**Table 1:** Characteristics of 19 FSW in Kampala, Uganda, 2022

Variable	N (%)
**Age**	
14–24	10 (57)
25–34	6 (32)
35–44	3 (16)
**Marital Status**	
Married	1 (5)
Not married	18 (95)
**Education Level**	
Primary	11 (58)
Secondary	7 (37)
Tertiary (degree)	1 (5)
**Duration on PrEP**	
<Six months	6 (32)
Six months to 1 year	4 (21)
One year to five years	8 (42)
>Five years	1 (5)

## Data Availability

The datasets generated and analyzed during the study are not publicly available in a repository; relevant data excerpts are in the manuscript. For researchers who meet the criteria for access to confidential data, their request will be evaluated on a case-by-case basis. Data inquiries may be directed to Ms. Ruth Mpirirwe at ruthmpirirwe@gmail.com.
